# Methicillin-resistant *Staphylococcus aureus* in Cat and Owner

**DOI:** 10.3201/eid1212.060725

**Published:** 2006-12

**Authors:** Carlo B. Vitale, T.L. Gross, J. Scott Weese

**Affiliations:** * San Francisco Veterinary Specialists, Inc., San Francisco, California, USA;; † California Dermatopathology Service and IDEXX Veterinary Services, West Sacramento, California, USA;; ‡ University of Guelph, Guelph, Ontario, Canada

**Keywords:** MRSA, cat, Methicillin-resistant Staphylococcus aureus

To the Editor: A 3-year-old, neutered male, domestic shorthaired cat was referred for treatment to a veterinary specialty clinic in San Francisco, California, with a 1-year history of multifocal patches of crusted and well-demarcated ulcers on the trunk. Initially, small crusts suspected to be associated with flea allergy and pyoderma were present; however, response was poor to multiple treatments, including repeated corticosteroid therapy and antimicrobial therapy with amoxicillin–clavulanic acid and enrofloxacin. The owner reported having skin abscesses and pneumonia 3 months earlier, although no microbiologic testing was performed.

Cytologic evaluation of exudate from the cat's lesions identified neutrophils and eosinophils with engulfed cocci. Leukocytosis with eosinophilia was found on a complete blood cell count. No notable abnormalities were present on thoracic radiograph, abdominal ultrasonograph, urinalysis, and tests for feline leukemia and immunodeficiency virus. Skin biopsy specimens were collected for histologic examination, and swabs of the exudates were submitted for bacterial culture. Histopathologic findings demonstrated ulcers and dermal granulation tissue with linearly arranged eosinophils, mast cells, neutrophils, and plasma cells between dense, homogeneous collagen bundles (sclerosing dermatitis). This pattern of inflammation is distinct from most staphylococcal infections of the skin, and it has been suggested that this uncommon histologic finding in cats is associated with methicillin-resistant staphylococcal infection (*1*).

Methicillin-resistant Staphylococcus aureus (MRSA) was isolated from the skin lesions. Identification was confirmed by detection of penicillin-binding protein 2a (PBP2a) by latex agglutination test (PBP2´ Test Kit, Oxoid, Hants, UK). Typing was performed by SmaI pulsed-field gel electrophoresis as previously described (*2*), and the isolate was classified as the USA300 clone. Genes encoding production of the Panton-Valentine leukocidin (PVL) were identified by real-time PCR (*3*). The isolate was susceptible to chloramphenicol, tetracycline, trimethoprim-sulfamethoxazole, and vancomycin, but resistant to β-lactams, enrofloxacin, and erythromycin. After identification of MRSA in the cat, swabs of the anterior nares were collected from the owner and the cat, and MRSA was identified in specimens from both. All isolates were indistinguishable.

This is the first report of isolation of USA300 MRSA from a household pet. USA300 is a community-associated clone that has disseminated widely throughout North America and Europe (*4**,**5*) and is reaching epidemic proportions in many regions. MRSA is becoming an important cause of skin and soft tissue infection in persons in the community (*4**,**5*) and has also been implicated in invasive infections such as necrotizing pneumonia (*6*). This clone possesses genes for PVL production, which may be an important factor in its apparent virulence (*4**,**5*). Additional characterization of the isolates from this study was not performed; however, USA300 has previously been reported to carry staphylococcal cassette chromosome mec (SCCmec) type IVa and classified as sequence type 8 (ST8) by multilocus sequence typing (*4**,**5*).

Reports of MRSA infection and colonization in pets have increased dramatically in the past few years (*3**,**7**–**9*). Although this rise may be partially the result of increased testing and reporting, MRSA is definitely emerging in pet populations throughout the world. The role of pets in transmission of MRSA is still unclear; however, recent evidence suggests that MRSA can be transmitted between persons and their pets, in both directions (*9**,**10*). Reports of MRSA infection and colonization in pets have indicated that pets tend to be infected with isolates that are consistent with clones that are predominant in the human population in their area (*7**–**9*). Accordingly, USA100 accounted for initial isolations of MRSA in pets in North America (*9*). The similarity between pet and human isolates has led to speculation that pet MRSA is closely linked to human MRSA and that the source of MRSA in pets may often be colonized humans. If this is the case, it is not surprising that USA300 would emerge as a cause of disease in pets as it increases in prevalence in the human population. Considering the rapid dissemination of USA300 in humans in the United States, particularly in California, where it is the predominant community-associated clone, finding USA300 in a household pet in that state is not unexpected.

Because indistinguishable isolates were collected from the owner and the infected cat, MRSA likely was transmitted between species in the household. However, while it is tempting to assume that the owner was the source of infection because of the owner's previous history of a soft tissue infection, this cannot be definitively determined on the basis of the timing of sampling in this case.

MRSA appears to be emerging as an important veterinary and zoonotic pathogen, and the epidemiology of MRSA in household pets may take a parallel course to that in humans. Ongoing MRSA surveillance in animals is required, including proper testing of specimens from clinically affected animals and surveillance for colonization. The potential for transmission of this clone between humans and pets should also be evaluated to clarify its epidemiology and to facilitate development of measures to reduce household transmission.
